# A Comparative Study of* Melissa officinalis* Leaves and Stems Ethanolic Extracts in terms of Antioxidant, Cytotoxic, and Antiproliferative Potential

**DOI:** 10.1155/2018/7860456

**Published:** 2018-05-16

**Authors:** Elena-Alina Moacă, Claudia Farcaş, Alexandra Ghiţu, Dorina Coricovac, Ramona Popovici, Nela-Loredana Cărăba-Meiţă, Florina Ardelean, Diana Simona Antal, Cristina Dehelean, Ştefana Avram

**Affiliations:** ^1^“Victor Babes” University of Medicine and Pharmacy Timisoara, Faculty of Pharmacy, Eftimie Murgu Square No. 2, RO-300041, Romania; ^2^University of Medicine and Pharmacy Targu Mures, Gheorghe Marinescu Street No. 38, RO-540139, Romania; ^3^“Victor Babes” University of Medicine and Pharmacy Timisoara, Faculty of Dentistry, Eftimie Murgu Square No. 2, RO-300041, Romania; ^4^University of Craiova, Faculty of Economics and Business Administration, Alexandru Ioan Cuza Street No. 13, RO-200764, Romania

## Abstract

*Melissa officinalis* L. has attracted an increased interest in recent years due to its multiple pharmacological effects. This study aimed to compare two* M. officinalis* ethanolic extracts, obtained from leaves and stems, with regard to their antioxidant activity, total phenolic content, and cytotoxic effects.* M. officinalis* ethanolic extracts showed a very good antioxidant activity in the DPPH test, correlated with the content in total phenols: higher in the case of* M. officinalis* from leaves extract (32.76 mg GAE/g) and lower for* M. officinalis* from stems extract (8.4 mg GAE/g). The lemon balm extracts exerted a cytotoxic effect on breast cancer cells (MDA-MB-231) even at low concentrations (100 *μ*g/mL), whereas, in the case of healthy HaCat cells,* M. officinalis* leaves extract only displayed cytotoxicity at much higher concentrations (500 and 1000 *μ*g/mL) and* M. officinalis* stems extracts were highly cytotoxic (starting at 100 *μ*g/mL). In addition, the extracts exerted inhibitory effects on cell migration and proliferation. These results provide information that confirms the high potential of* M. officinalis* as a source of chemopreventive agents. Moreover, these data can be considered a solid background for further* in vivo* studies involving mice bearing breast tumors.

## 1. Introduction

*Melissa officinalis* is a perennial herb, member of the Lamiaceae family, known under multiple names, such as bee balm, lemon balm, honey balm, melissa, and garden balm. The use of* M. officinalis* as remedy in traditional medicine dates from many years and several inquiries have been conducted to identify its healing properties [1–8].* M. officinalis* has an average height of 30–125 cm and displays ovate, dark-green leaves and white flowers which bloom in the summer [9, 10].* M. officinalis* has a large use in traditional medicine, food industry, and aromatherapy, due to its fresh smell and its medicinal properties including hypoglycemic, hepatoprotective, antimicrobial, antidepressant, hypnotic, and sedative [1, 11–14]. In addition, there are studies that pointed out the cytotoxic effect of lemon balm extract on breast cancer [13] and colon carcinoma [15]. Chemical composition of lemon balm is diverse and includes phenolic acids, tannins, flavonoids, terpenes (triterpenes, monoterpenes, and sesquiterpenes), and volatile compounds. The main active ingredients comprise volatile compounds: neral, geranial, citronellal, and geraniol, phenolic compounds: luteolin, caffeic acid, hesperidin, naringin, coumarinic acid, and rosmarinic acid, and triterpenes: oleanolic and ursolic acid [10].

Not only does lemon balm have an impressive background from traditional medicine, but, according to the latest studies, it is effective in cardiovascular disease, by decreasing the values of total lipid concentration, improving HDL (high density lipoprotein) values, and lowering the hepatic synthesis of cholesterol [13].

Lemon balm exerts, as well, an antioxidant effect [16] and is involved in the pituitary gland function by improving the hormonal levels of TSH (thyroid-stimulating hormone), T3 (triiodothyronine), and T4 (thyroxine) [13]. Some studies showed that the extract of* M. officinalis* has anti-inflammatory and antinociceptive effect by interacting with muscarinic and nicotinic receptors. Due to the volatile compounds,* M. officinalis* can be used in gastric diseases, by helping the digestion and calming the spasms of the abdominal muscle [1].

Lin and coworkers [17] have studied the composition and the antioxidant and antiproliferative effect of* M. officinalis* extracts. Their studies provided information regarding the composition of the extract of lemon balm and the fact that hot air drying technique is less effective than freeze drying technique because of the losses of phytochemical substances. The authors showed that both types of extracts: freeze dried and hot air dried, displayed antiproliferative activities on the studied cells (HepG2, KB-epidermal carcinoma of the mouth, and TSGH 9201-gastric adenocarcinoma), in a concentration-dependent manner, and regarding the antioxidant effect, the freeze dried extract had higher activity than the hot air extract.

Encalada and coworkers [18] proved the existence of a direct correlation between the phenolic content of* M. officinalis*, the antioxidant effect, and the antiproliferative effect. In addition, it was thought that the content of rosmarinic acid is responsible for the cytotoxic effect in colon human cancer cell lines tested. Based on these data, it was considered that the vegetal extract of* M. officinalis* could represent a good medical strategy in human colon cancer, alongside the cytotoxic drugs.

Most studies are focused on the leaves of the lemon balm, which are the plant product included in pharmacopoeias and which contain a high number of secretory glands. However, more recently, stems and whole aerial parts were as well considered for research, in order to increase the valorization of all herbal material including less common indications [19, 20]. In this idea, our study performs a comparative evaluation of leaves and stems extracts. It undertakes the assessment of the antioxidant activities, the total phenolic contents, and the cytotoxic and the antiproliferative effects of* M. officinalis *extracts obtained from both types of plant organs.

## 2. Experimental Part

### 2.1. Materials

#### 2.1.1. Chemicals and Reagents

The reagents used for obtaining the extracts: ethanol 96% (v/v), distilled water, 2,2-diphenyl-1-picrylhydrazyl (DPPH) (Batch No: # STBF5255V), and gallic acid, were purchased from Chemical Company SA, Iasi, Romania, and Sigma Aldrich (Germany), respectively. The ascorbic acid was acquired from Lach-Ner Company (Czech Republic) and the Folin-Ciocalteu reagent from Scharlau (Spain). The chemicals used for cell culture: Dulbecco's modified Eagle Medium (DMEM) high glucose, trypsin/EDTA solution, phosphate saline buffer (PBS), penicillin/streptomycin solution, fetal bovine serum (FBS), Trypan blue, and Alamar blue solutions, were supplied by Sigma Aldrich (Germany) and Thermo Fisher Scientific (USA).

#### 2.1.2. Cell Lines

The cell lines used in the present study: breast adenocarcinoma cells (MDA-MB-231) and human immortalized keratinocytes (HaCat), were acquired from ATCC (American Type Culture Collection) as frozen vials.

#### 2.1.3. Plant Material

Plant samples,* Melissa officinalis *leaves and stems, were collected from the Botanical garden of “Victor Babes” University of Medicine and Pharmacy Timisoara and identified by the Department of Pharmaceutical Botany. Voucher no. 125/20016 was kept in the mentioned department. After collection, the samples were dried at room temperature and stored in proper conditions.

### 2.2. Methods

#### 2.2.1. *Melissa officinalis* Ethanolic Extracts Preparation

**Figure 1:**
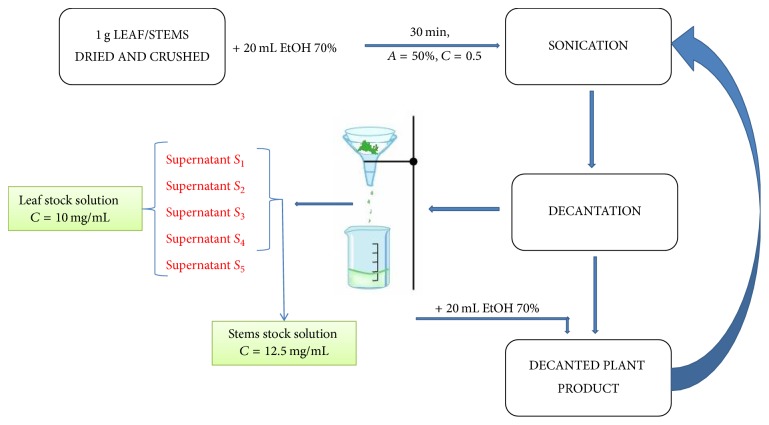
Schematic protocol of* M. officinalis* ethanolic extracts preparation.

The two ethanolic extracts were obtained according to the method of Kamdem et al. [21], slightly modified. The ethanolic extracts of* M. officinalis* leaves and stems were obtained as follows: 1 g of dried and crushed leaves was mixed with 20 mL ethanol 70% and sonicated for 30 minutes at room temperature (amplitude *A* = 50% and cycle *C* = 0.5). After sonication the sample was filtered, yielding supernatant *S*_1_. The plant residue was mixed again with 20 mL ethanol 70%, and the sonication procedure was repeated at the same parameters and in the same conditions, obtaining supernatant *S*_2_. The procedure was repeated three more times, and the supernatants *S*_1_–*S*_5_ were reunited, yielding the* M. officinalis* stock solution obtained from leaves. The ethanolic extract of the* M. officinalis* stems was obtained by the same procedure, with the difference that the process was repeated only four times yielding* M. officinalis* stock solution obtained from stems. Thus, two types of ethanolic extracts were prepared from leaves (stock concentration: 10 mg/mL) and stems (stock concentration: 12.5 mg/mL) of* M. officinalis*, the schematic protocol being pictured in Figure 1.

#### 2.2.2. Antioxidant Activity Assay

The antioxidant activity of* M. officinalis* extracts was determined by DPPH- (2,2-diphenyl-1-picrylhydrazyl-) free radical scavenging assay. DPPH-free radical scavenging capacity of the extracts was evaluated according to the method of Manzocco et al. [22]. Briefly, an amount of 0.5 mL of each extract was added to 2 mL solvent (ethanol 70%, v/v) and to 0.5 mL ethanol solution of DPPH radical 1 mM. The absorbance of each sample (*A*_sample_), including the absorbance of DPPH and ascorbic acid, was measured for 20 minutes continuously, from 5 to 5 seconds, with a T70 UV/VIS Spectrophotometer (PG Instruments Ltd.) at 516 nm. This mode of analysis allows us to determine the reaction speed by which the DPPH is consumed by the antioxidants present in the sample.

The percent of antioxidant activity (% AOA) of the samples was calculated with the following formula:

(1) $$\begin{array}{c} \%\;\;AOA=\left[\;100-\left(\;\frac{A_{sample}}{A_{DPPH}}\;\right)\times 100\;\right], \end{array}$$

where

  AOA is antioxidant activity;
 
*A*_sample_ is absorbance of the sample;
 
*A*_DPPH_ is absorbance of DPPH.

The antioxidant activities of the lemon balm extracts were compared to that of ascorbic acid (0.1 mg/mL in ethanol 96% v/v) used as control.

#### 2.2.3. Determination of Total Phenolic Content (TPC)

The total phenolic content (TPC) of the lemon balm extract was estimated by the Folin-Ciocalteu method [23] using gallic acid (0.1–1 mg/mL) as a standard. This method is based on electron transfer reactions between Folin-Ciocalteu reagent and phenolic compounds, when a blue colored complex that can be quantified spectrophotometrically is formed [23, 24].

In brief, Folin-Ciocalteu reagent (diluted 1 : 10; 2.5 mL) was mixed with plant extract (0.5 mL) and Na_2_CO_3_ 7.5% (2 mL). The mixture was incubated for 2 hours in the dark at room temperature. Subsequently, absorbance was measured at 750 nm, using a T70 UV/VIS Spectrophotometer (PG Instruments Ltd.). The total phenolic content was expressed as gallic acid equivalents (mg of GAE/g sample). Calibration range of gallic acid was from 0.1 to 1 mg/mL.

#### 2.2.4. Cell Culture

Cells were cultured in Dulbecco's Modified Eagle's Medium (DMEM) high glucose (4.5 g/L) supplemented with 10% fetal bovine serum (FBS) and 1% Penicillin/Streptomycin mixture. The cells were maintained in standard conditions, in a humidified atmosphere at 37°C and 5% CO_2_ in a Steri-Cycle i160 incubator (Thermo Fisher Scientific, USA). Both cell lines were grown in 75 cm^2^ culture flasks and were routinely passaged every 2-3 days, by treatment with trypsin-EDTA 0.25%, after attaining 80–85% confluence. Cell counting was performed with Countess™ II Automated Cell Counter, in Trypan blue presence. All* in vitro* studies were performed under sterile conditions by using a Biological Safety Cabinet, MSC Advantage 12 model (Thermo Fisher Scientific, USA).

#### 2.2.5. Cell Viability Assay: Alamar Blue

The cytotoxic activity of both* M. officinalis* ethanolic extracts was evaluated on the two cell lines (HaCat and MDA-MB-231) by using Alamar blue (AB) viability test. The AB assay is based on a colorimetric reaction in which resazurin (dark blue compound) is converted by mitochondrial reductase of viable cells to resorufin (a pink compound with an intense fluorescence).

Briefly, 1 × 10^4^ cells/well were seeded onto a 96-well plate. After 24 h incubation time, cell media was removed and the attached cells were stimulated with five different concentrations of* M. officinalis* extracts (20, 100, 250, 500, and 1000 *μ*g/mL) for 24 h. Control cells were stimulated with the same amount of ethanol 70%, as the amount of extract stock solutions added. In order to determine cells viability, the absorbance was measured spectrophotometrically at 570 nm and 600 nm using a microplate reader (xMark™ Microplate, Biorad). Wells containing medium without cells were used as negative controls and the results were calculated according to a previous published formula [25, 26].

#### 2.2.6. Antimigratory Potential: Wound Healing Assay Method

For the determination of a possible inhibitory effect of* M. officinalis* extracts on migration and invasion of both cell lines (MDA-MB-231 and HaCat), the scratch assay test was applied. It is a well-known, practical, and easy method to express cell to cell interactions. The principle of this technique consists of drawing a scratch on the middle of the well, by using a small sterile pipette tip (10 *μ*l) and supervising the gap filling by taking pictures at different time points (0, 3, and 24 h).

A number of 2 × 10^5^ cells/well were seeded onto 12-well culture plates until a 90% confluence was reached. After that, the gaps/scratches were drawn in each well using a sterile pipette tip. The detached cells were removed by gently washing the plate with PBS. After this step, cells were stimulated with both* M. officinalis* extracts using three different concentrations: 20, 100, and 250 *μ*g/mL, and as control the same amount of alcohol as the volume of stock solutions used for preparation of the* M. officinalis* extracts samples was added. Photos were taken at 0, 3, and 24 h with the Olympus IX73 inverted microscope and the cellSense Dimension software was used to analyze the dimension of the gap.

#### 2.2.7. Statistical Analysis

The statistical programs and software programs applied in the present study were GraphPad Prism 5 and Origin 8 (Origin Lab—Data analysis and Graphing Software). The results were expressed as the mean ± standard deviation. One-way ANOVA analysis was applied to determine the statistical differences followed by Tukey posttest (^*∗*^*p* < 0.05; ^*∗∗*^*p* < 0.01; ^*∗∗∗*^*p* < 0.001).

## 3. Results and Discussion

### 3.1. Antioxidant Activity Assay

**Figure 2:**
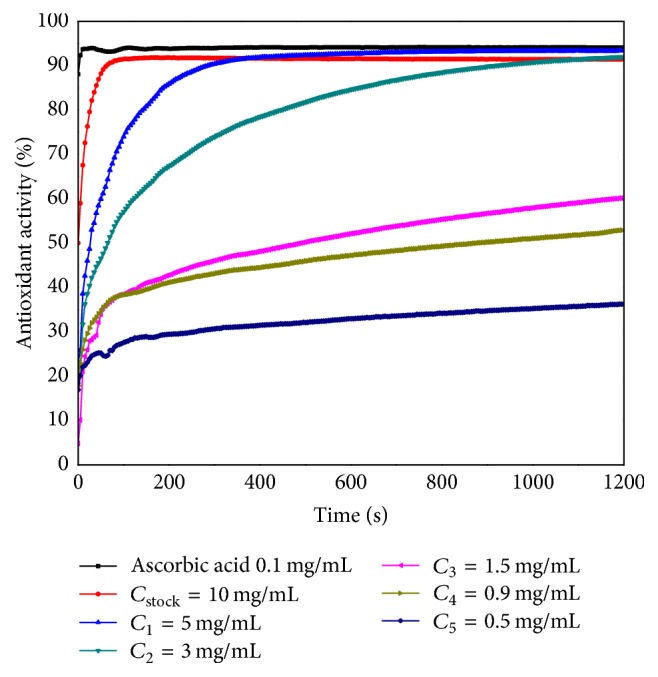
The time-dependent antioxidant activity of the* Melissa officinalis* leaves ethanolic extract.

The antioxidant activity (AOA) of the* M. officinalis* stock solution (*C*_stock_ = 10 mg/mL) obtained from leaves and its five dilutions (*C*_1_ = 5 mg/mL; *C*_2_ = 3 mg/mL; *C*_3_ = 1.5 mg/mL; *C*_4_ = 0.9 mg/mL; *C*_5_ = 0.5 mg/mL) were recorded in a time-dependent manner over 1200 seconds and presented in comparison with ascorbic acid AOA, which was considered the positive control (Figure 2).

The stock solution of* M. officinalis* leaves showed a high antioxidant activity. The ethanolic extract reacted very quickly with DPPH radical, scavenging the substrate in the first 100 seconds. The antioxidant compounds present in the ethanolic extract of 5 mg/mL (*C*_1_) consumed the DPPH radical in 400 seconds, whereupon the absorbance of the sample slightly decreased, leading to an increased AOA in comparison to that of the stock solution, being very similar to the AOA of ascorbic acid.

Regarding the subsequent dilutions *C*_2_ = 3 mg/mL to *C*_5_ = 0.5 mg/mL, the compounds from the samples reacted with DPPH throughout the recording time of the analysis; all investigated solutions proved to have an antioxidant activity. The equilibrium was not reached even after 1200 seconds (20 minutes). The AOA of the ethanolic extract of 3 mg/mL (*C*_2_) attained similar values with the stock solution (*C*_stock_ = 10 mg/mL) extract after 1100 seconds. At the initial moment the AOA values were much lower than the ones recorded at the final moment, after 20 minutes of analysis. Based on these results, it can be concluded that the antioxidant activity of the samples was concentration-dependent. These data are in accordance with the literature [27, 28].

**Figure 3:**
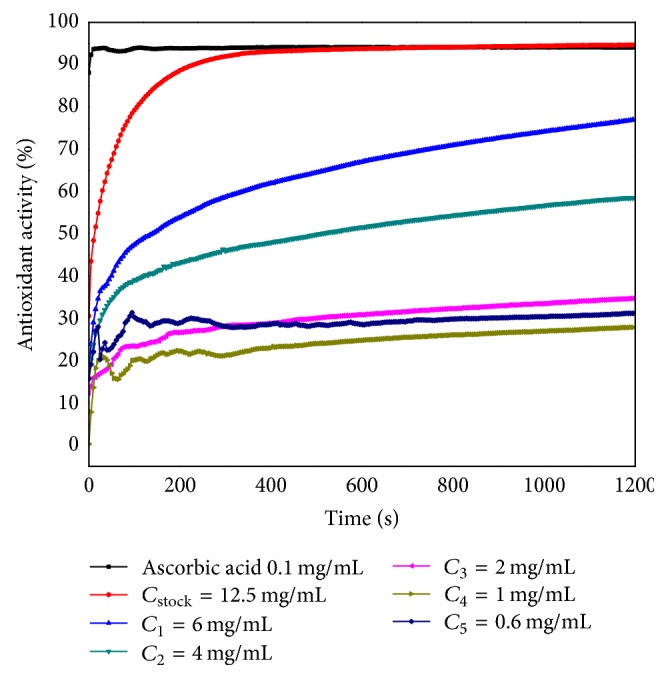
The time-dependent antioxidant activity of the* M. officinalis* stems ethanolic extract.

The AOA for the ethanolic extract obtained from* M. officinalis* stems was also determined: stock solution (*C*_stock_ = 12.5 mg/mL) and other five dilutions: *C*_1_ = 6 mg/mL, *C*_2_ = 4 mg/mL, *C*_3_ = 2 mg/mL, *C*_4_ = 1 mg/mL, and *C*_5_ = 0.6 mg/mL (Figure 3). The values recorded in time were compared with the AOA of the ascorbic acid.

The highest AOA (attained after 1200 seconds) was displayed by the stock solution, the value being slightly higher than the value recorded for ascorbic acid, although the reaction was much slower than that for ascorbic acid. The components of the* M. officinalis* stems extract used up the DPPH radical after approximately 300 seconds, followed by reaching the reaction equilibrium.

Regarding the dilutions of the stock solution, their kinetic was similar to the one of* M. officinalis* leaves solutions, and equilibrium was not reached even after 20 minutes. Moreover, the first two diluted samples (*C*_1_ = 6 mg/mL and *C*_2_ = 4 mg/mL) displayed fluctuations of the antioxidant activity; namely, they reacted faster with DPPH in the first 400 seconds. This phenomenon is probably due to the relatively low amounts of antioxidant compounds in the stems. The antioxidant activity of the* M. officinalis* stems solutions was as well concentration-dependent, similar to the leaves extract.

Our findings are in accordance with the data described in the literature: the compound considered to be responsible for the antioxidant effect is rosmarinic acid, a phenylpropanoid derivative which is present in all organs of the* M. officinalis* plant [29]. It was established that the antioxidant effect of* M. officinalis* extract is up to ten times stronger than the effect of vitamin C [28, 30]. The* M. officinalis* extract exhibits also antinociceptive and anti-inflammatory effects, which are ascribable to rosmarinic acid as well as to terpenoids and flavonoids present in the extract [31].

In Table 1 is displayed the percent of inhibition (AOA) induced by ascorbic acid,* M. officinalis* leaves, and stems ethanolic extracts.

The inhibition percent was calculated from the following equation:

(2) $$\begin{array}{c} \%\;\;inhibition=\frac{\left(A_{control}-A_{sample}\right)}{A_{control}}\cdot 100 \end{array}$$

The IC_50_^DPPH^ values for ascorbic acid (IC_50_ = 3.22 · 10^−6^  ± 0.0002 mg/mL),* M. officinalis* leaves (IC_50_ = 0.66 ± 0.01 mg/mL), and stems (IC_50_ = 10.27 ± 0.45 mg/mL) extracts were also calculated. IC_50_^DPPH^ value represents the concentration of the antioxidant compounds from each sample required to scavenge DPPH radical by 50%. The IC_50_^DPPH^ parameter was determined using GraphPad Prism 5 software.

Koksal and coworkers evaluated the antioxidant activity of two extracts of* M. officinalis* (aqueous and ethanolic), using the DPPH method. They proved that the water extract of* M. officinalis* has effective antioxidant and radical scavenging activities as compared to ethanolic extract. The IC_50_ values for both water (31.4 *μ*g/mL) and ethanolic extract (202.7 *μ*g/mL) were, also, established, data that are in agreement with our results [32].

*M. officinalis* proved to have also anxiolytic effect and circadian activities, due to significant amounts of rosmarinic acid, oleanolic acid, ursolic acid, and triterpenoids [33]. Previous studies reported by our research group indicated that triterpenes are potent antioxidant and antitumor compounds [34].

Pereira and coworkers have demonstrated that* M. officinalis* extract asserts prevention against some diseases associated with oxidative stress, due to the antioxidant activity of phenolic compounds such as quercetin, garlic acid, quercitrin, and rutin [16].

### 3.2. Total Phenolic Content (TPC)

Depending on the plant part used for preparation (leaves or stems), the content of total phenols varied with one order of magnitude. The TPC in the leaves extract was 32.76 mg GAE/g dry material, while, in the extract from stems, TPC was much lower (8.4 mg GAE/g dry material). Studies evaluating the TPC of lemon balm extracts show an important variation of results. For example, the methanolic extract obtained from the aerial parts of* M. officinalis* growing in Romania had a TPC of 22 mg GAE/g extract [35]. On the contrary, for the methanolic extract of plant material from Bulgaria (herb) a TPC of 48.86 mg GAE/100 g dry weight was measured [36]. The variation of TPC values according to the extraction method and the solvent used is also reported in the literature. For the extract obtained by maceration, the TPC content was 90.1 mg GAE/g dry material, whereas, for the extract prepared by the means of ultrasounds, TPC was 105.5 mg GAE/g dry material [37].

The phenolic content of lemon balm extract is also influenced by the solvent used for its preparation. A high phenolic content was estimated for the hydroalcoholic extract from lemon balm leaves (227.6 mg GAE/g dry material) [38].

Phenolic compounds have been investigated in numerous studies for their antioxidant activity which is important in the prevention and therapy of several diseases, including cancer [39]. Previous studies proved that polyphenolic compounds determined a strong antioxidant activity that might influence the biological response [34, 40].

### 3.3. Cytotoxicity Assessment by the Means of Alamar Blue

One of the aims of the present study consisted in verifying the cytotoxic effect of the extracts on normal (human keratinocytes, HaCat) and breast cancer (MDA-MB-231) cell lines. Five different concentrations were tested (20, 100, 250, 500, and 1000 *μ*g/mL) for 24 h. Our results indicated that the solvent (ethanol 70%) induced a cytotoxic effect by itself even at the lowest concentration (20 *μ*g/mL) as compared to control cells (unstimulated), this being the reason why the cytotoxicity data were reported to the solvent effect.

**Figure 4:**
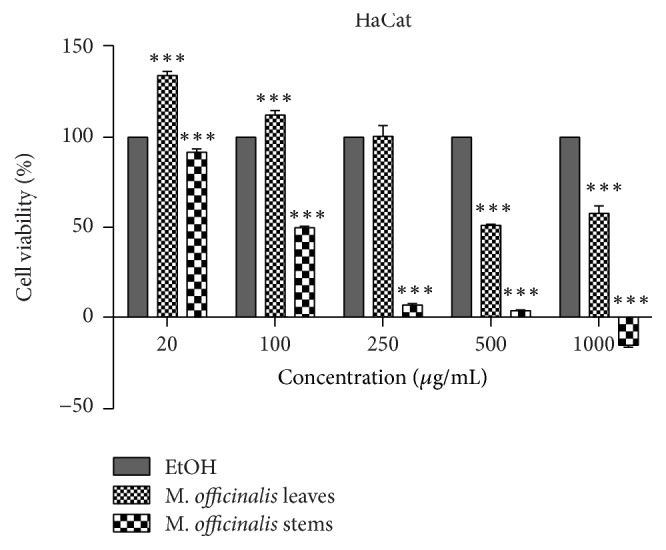
Percentage of viable cells—human keratinocytes (HaCat), after stimulation with* M. officinalis* leaves and stems extracts (20, 100, 250, 500, and 1000 *μ*g/mL) for 24 h. The viability was assessed by using Alamar blue assay and was expressed as percentage of viable cells (%) related to positive control (cells stimulated with EtOH, the solvent used for obtaining the extracts). Data are expressed as mean values ± SD of three independent experiments done in triplicate and ^*∗∗∗*^*p* < 0.001 and were calculated by one-way ANOVA followed by Tukey's post hoc test.

The impact of the two types of* M. officinalis* extracts—leaves and stems—on normal human keratinocytes viability is presented in Figure 4.

Figure 4 indicates that the* M. officinalis* leaves' ethanolic extract induced a dose-dependent cytotoxicity (IC_50_ = 301.4 ± 10.26 *μ*g/mL), the highest concentrations tested (500 and 1000 *μ*g/mL) being toxic for HaCat cells when compared to solvent, whereas the low concentrations (20, 100, and 250 *μ*g/mL) were devoid of toxicity.

In the case of* M. officinalis* stems extracts (Figure 4), the situation was quite different, the cytotoxic effect being observed at concentration of 100 *μ*g/mL (IC_50_ = 109.63 ± 7.68 *μ*g/mL). After stimulation with 250 and 500 *μ*g/mL, the percentage of viable cells recorded was lower than 10%, and the highest concentration tested (1000 *μ*g/mL) induced a negative rate.

**Figure 5:**
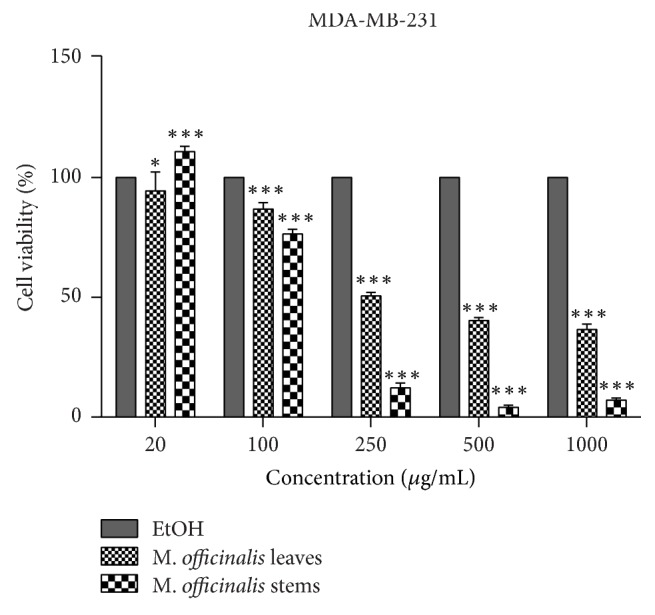
Percentage of viable cells—breast cancer cells (MDA-MB-231), after stimulation with* M. officinalis* leaves and stems extracts (20, 100, 250, 500, and 1000 *μ*g/mL) for 24 h. The viability was assessed by using Alamar blue assay and was expressed as percentage of viable cells (%) related to positive control (cells stimulated with EtOH, the solvent used for obtaining the extracts). Data are expressed as mean values ± SD of three independent experiments done in triplicate and ^*∗∗∗*^*p* < 0.001 and ^*∗*^*p* < 0.05 and were calculated by one-way ANOVA followed by Tukey's post hoc test.

The effect of the two extracts on breast cancer cells (MDA-MB-231) was also verified (Figure 5).* M. officinalis* leaves' extract induced a cytotoxic effect starting with 100 *μ*g/mL (IC_50_ = 196.66 ± 13.93 *μ*g/mL), the most significant cytotoxic effects being recorded for 500 and 1000 *μ*g/mL (Figure 5).

*M. officinalis* stems' extract exerted a stronger cytotoxic effect on MDA-MB-231 cells (Figure 5) as compared to the* M. officinalis* leaves extract, the percentage of viable cells being more reduced for most of the concentrations tested (IC_50_ = 103.73 ± 6.69 *μ*g/mL), the strongest cytotoxic effect being induced by the 500 *μ*g/mL concentration (4.08%).

Based on the results obtained, it could be stated that the active principles of both* M. officinalis* ethanolic extracts exerted antitumor activity by reducing the viability of breast adenocarcinoma cells at concentrations higher than 100 *μ*g/mL.

Similar results were described by Saraydin and coworkers when an aqueous* M. officinalis* extract was tested on different breast cancer cells (MCF-7, MDA-MB-231, and MDA-MB-468) [41]. The cytotoxic effects of* M. officinalis *were also proved on human colon cancer cells—HCT-116 [18]. A recent study showed that, at a concentration of 50 *μ*g/mL, an ethanolic extract of* M. officinalis *induced a low growth inhibition percentage (under 15%) to a panel of human cancer cell lines (melanoma, breast cancer, colon cancer, osteosarcoma, leukemia, etc.) [42]. Jahanban-Esfahlan and collaborators proved the antiproliferative properties of a hydroalcoholic* M. officinalis* extract on lung (A549), breast (MCF-7), ovarian (SKOV3), and prostate (PC-3) cancer cells, the cytotoxic effect being tumor type specific [40].

### 3.4. The Antimigratory Effect via Wound Healing Assay Method

**Figure 6:**
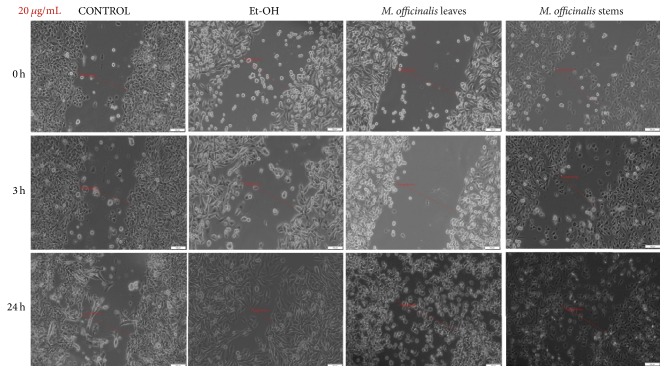
The effect of* M. officinalis* ethanolic extracts (20 *μ*g/mL) on HaCat cells' migration and proliferation. The pictures were taken at 0, 3, and 24 h after stimulation.

The ability of cancer cells to migrate is well-known as well as the key role of this process in tumor progression and metastasis. In order to verify the antimigratory effect of the* M. officinalis* leaves and stems extracts by the means of scratch assay, the smallest concentrations were tested (20, 100, and 250 *μ*g/mL), concentrations that proved to be less cytotoxic for the cells. It can be observed in Figure 6 that* M. officinalis* stems extract, at the concentration of 20 *μ*g/mL, exhibited a superior antimigratory effect on HaCat cells at 24 hours after stimulation, when compared with* M. officinalis* leaves extract at the same concentration.

**Figure 7:**
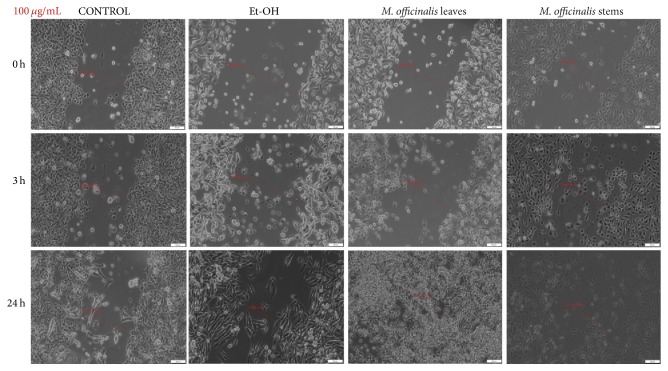
The effect of* M. officinalis* ethanolic extracts (100 *μ*g/mL) on HaCat cells' migration and proliferation. The pictures were taken at 0, 3, and 24 h after stimulation.

As showed in Figure 7, both* M. officinalis* extracts at the concentration of 100 *μ*g/mL manifested antimigratory effect, at 3 h after stimulation. After 24 h, HaCat cells became detached and changed their shape and morphology, the most significant changes being observed after* M. officinalis* leaves extract stimulation. In addition, pictures indicated that ethanol (solvent) at concentration 100 *μ*g/mL did not affect migration and proliferation of HaCat cells (Figure 7).

**Figure 8:**
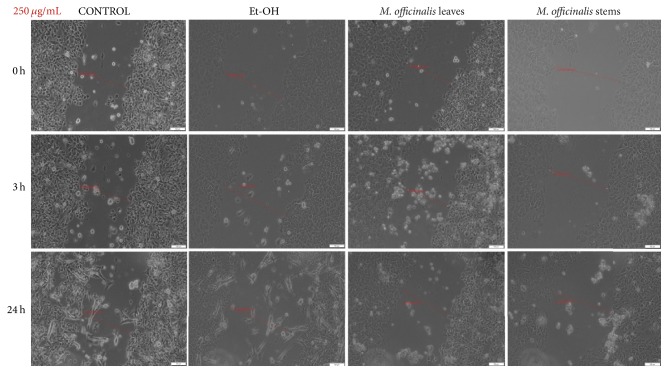
The effect of* M. officinalis* ethanolic extracts (250 *μ*g/mL) on HaCat cells' migration and proliferation. The pictures were taken at 0, 3, and 24 h after stimulation.

Stimulation of HaCat cells with 250 *μ*g/mL of* M. officinalis* ethanolic extracts led to a significant antimigratory effect observed after 3 h of stimulation (Figure 8). Moreover, as it can be seen in the pictures, even at 3 h after stimulation there were detached cells that were floating in the medium, with round shape characteristic to apoptotic cells, the number of these cells becoming higher at 24 h after stimulation, indicating the cytotoxic properties of these extracts, data that are consistent with the results obtained by cell viability assay. At 24 h after stimulation, the shape and the morphology of the cells that were attached to the plate seemed to be modified. The ethanol tested in the same concentration (250 *μ*g/mL) as positive control did not have a negative impact on proliferation of keratinocyte cell line 24 h after stimulation (Figure 8).

**Figure 9:**
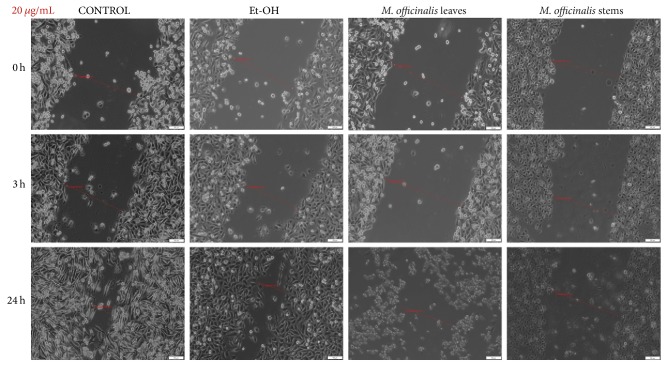
The effect of* M. officinalis* ethanolic extracts (20 *μ*g/mL) on MDA-MB-231 cells' migration and proliferation. The pictures were taken at 0, 3, and 24 h after stimulation.

**Figure 10:**
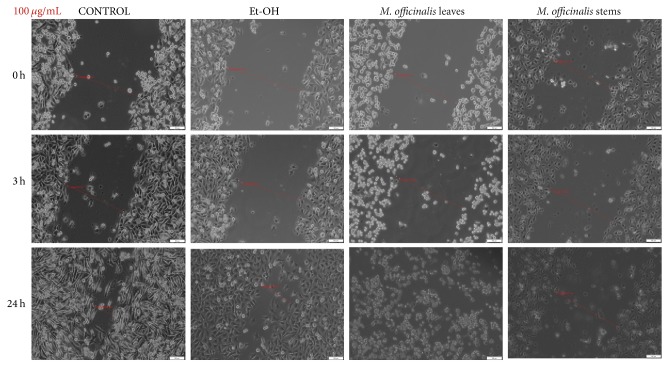
The effect of* M. officinalis* ethanolic extracts (100 *μ*g/mL) on MDA-MB-231 cells' migration and proliferation. The pictures were taken at 0, 3, and 24 h after stimulation.

**Figure 11:**
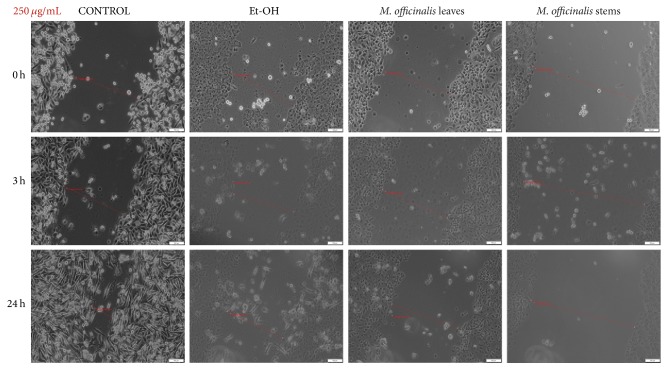
The effect of* M. officinalis* ethanolic extracts (250 *μ*g/mL) on MDA-MB-231 cells' migration and proliferation. The pictures were taken at 0, 3, and 24 h after stimulation.

*M. officinalis* ethanolic extracts' stimulation revealed an inhibitory effect on MDA-MB-231 cells migration and proliferation (Figures 9–11). From the comparison of control pictures, it can be observed that MDA-MB-231 breast adenocarcinoma cells possess invasive properties, the created gaps becoming filled by cells at 24 h (the size range of the gaps varied from 488.23 *μ*m at 0 h to 169.82 *μ*m at 24 h). The use of ethanol as positive control did not influence the migration and proliferation of MDA-MB-231 cells at 20 and 100 *μ*g/mL (Figures 9 and 10), whereas at 250 *μ*g/mL some cells were observed floating in the medium at 24 h (Figure 11).

The antimigratory effects of both* M. officinalis* extracts were detected from 3 h after stimulation with all concentrations (20, 100, and 250 *μ*g/mL). Changes in the breast cancer cells shape and morphology could be observed after 24 h, even at the lowest concentration tested (20 *μ*g/mL). These effects were more pregnant after stimulation with* M. officinalis* leaves extract (Figures 9–11). The presence of detached and round cells after stimulation indicated the cytotoxic effect of these extracts, results that are in agreement with the ones recorded by the means of Alamar blue assay.

The* in vitro* tests performed in the present study denoted that both* M. officinalis* ethanolic extracts exerted cytotoxic effects on healthy cells (only at the highest concentrations—250, 500, and 1000 *μ*g/mL), but also, on breast cancer cells, the cytotoxicity being induced starting with 100 *μ*g/mL, the strongest cytotoxic effect was exhibited by the* M. officinalis* stems extract. In addition, the lowest concentrations of* M. officinalis* ethanolic extracts (20, 100, and 250 *μ*g/mL) determined an inhibitory effect on migration and proliferation of both types of cells.

According to previous studies,* M. officinalis* extract was tested on colon cancer cells and breast cancer cells and proved to be cytotoxic.* M. officinalis* alcoholic extract induced apoptosis and inhibited proliferation of the colon cancer cells through generation of reactive species of oxygen [15]. Moreover, it was established that rosmarinic acid from* M. officinalis* extract was responsible for the antimigratory effect of colon cancer cells HCT-116 [18].

*M. officinalis* extract proved to have antiproliferative activity even on hormone-dependent cells [40]. The study accomplished by Saraydin and coworkers [41] on breast cancer cells (MCF-7, MDA-MB-468, and MDA-MB-231) corroborates our results, according to which* M. officinalis* extract may represent the therapeutic alternative in chemoprevention of breast cancer. Nevertheless, it is imperiously necessary for these effects to be sustained by* in vivo* studies.

The main observation is that antiproliferative and antioxidant activity are increased in a direct relation with extract concentration.* M. officinalis* ethanolic extracts developed a positive effective/toxic relationship on human breast carcinoma MDA-MB-231* in vitro*. Anyway the concentrations applied on experiments were not very increased and this led to the idea that* M. officinalis* ethanolic extracts are very active in human breast carcinoma data that are related to other reported information [41].

## 4. Conclusions

The present study undertakes an evaluation of cytotoxic and antiproliferative effects of extracts obtained from the leaves and stems of lemon balm, with a special focus on breast cancer cells (MDA-MB-231). These extracts are characterized with regard to their content in total phenolic compounds and antioxidant activities; the extracts from leaves display a higher content in antioxidant phytocompounds and hence a higher bioactivity against free radicals. Both extracts from leaves and from stems demonstrated relevant cytotoxic effects. However, their profile is different. While leaf extracts are selectively more cytotoxic against cancer cells than against normal HaCat cells, stem extracts have a higher cytotoxic activity against MDA-MB-231 cells but displayed a lower selectivity when compared to healthy cells. The ratio of activity and toxicity for* M. officinalis* extracts is positive and the antitumor activity is significant. These data represent the background for further* in vitro* studies required to elucidate the antitumor mechanism of action.

## Figures and Tables

**Table 1:** The percent of inhibition (AOA) induced by ascorbic acid as compared to *M. officinalis* leaves and stems ethanolic extracts.

Ascorbic acid		*M. officinalis* leaves		*M. officinalis* stems	
Concentration [mg/mL]	% inhibition	Concentration [mg/mL]	% inhibition	Concentration [mg/mL]	% inhibition
0.1	94.17	10	91.43	12.5	94.68
0.08	94.65	5	93.36	6	76.53
0.06	94.23	3	91.96	4	58.20
0.04	93.99	1.5	59.69	2	34.73
0.02	80.67	0.9	52.22	1	27.82
0.005	27.48	0.5	36.22	0.6	31.21
